# Whole-Genome Sequencing Analysis of Antimicrobial Resistance, Virulence Factors, and Genetic Diversity of *Salmonella* from Wenzhou, China

**DOI:** 10.3390/microorganisms12112166

**Published:** 2024-10-27

**Authors:** Yafang Jin, Yi Li, Shaojie Huang, Chengji Hong, Xucong Feng, Huidi Cai, Yanmei Xia, Shengkai Li, Leyi Zhang, Yongliang Lou, Wanchun Guan

**Affiliations:** 1 Wenzhou Key Laboratory of Sanitary Microbiology, Key Laboratory of Laboratory, Medicine, Ministry of Education, School of Laboratory Medicine and Life Sciences, Wenzhou Medical University, Wenzhou 325035, China; jinyafang1991@foxmail.com (Y.J.); huangsj2021@foxmail.com (S.H.); fengxucong@foxmail.com (X.F.); caihuidi@foxmail.com (H.C.); xia3031946566@foxmail.com (Y.X.); zxzzzhu@gmail.com (S.L.); 2 Institute of Marine Science, Wenzhou Medical University, Wenzhou 325035, China; 3 Wenzhou Center for Disease Control and Prevention, Wenzhou 325035, China; zjwzliyi@126.com (Y.L.); hongcjcdc@126.com (C.H.); zhleyi@126.com (L.Z.)

**Keywords:** *Salmonella*, antimicrobial resistance, *Salmonella* pathogenicity islands, cgMLST, whole-genome sequencing

## Abstract

*Salmonella* species are important foodborne pathogens worldwide. *Salmonella* pathogenicity is associated with multiple virulence factors and enhanced antimicrobial resistance. To determine the molecular characteristics and genetic correlations of *Salmonella*, 24 strains of *Salmonella* isolated from different sources (raw poultry, human stool, and food) in the Wenzhou area were investigated to determine the distribution of antimicrobial resistance and virulence determinants using whole-genome sequencing (WGS). Aminoglycoside resistance genes were detected in all samples. Over half of the samples found antimicrobial resistance genes (ARGs) and point mutations for several clinically frequently used antibiotic, beta-lactams, tetracyclines, and quinolones. Of these strains, 62.5% were predicted to be multidrug-resistant (MDR). The quinolone-modifying enzyme gene *aac(6’)-Ib-cr*, detected in five samples (S1–S4 and S10), was located on integrons. The analysis of *Salmonella* pathogenicity island (SPI) profiles suggests that serotypes with close genetic relationships share the same distribution of virulence factors, revealing a link between genotype and SPI profiles. cgMLST analysis indicated that five isolates S14–S18 were closely related to strains originating from the United Kingdom, suggesting that they may share a common origin. Data from this study may enrich the molecular traceability database for *Salmonella* and provide a basis for effective public health policies.

## 1. Introduction

*Salmonella* is a facultative anaerobic Gram-negative bacteria belonging to the *Enterobacteriaceae* family. It is an important zoonotic foodborne pathogen found in animal products such as meat of poultry, pork, and eggs [1,2]. More than 90 million infections and 150,000 deaths are recorded each year due to the consumption of *Salmonella*-contaminated meat worldwide [3]. *Salmonella* is the second most common bacterial cause of foodborne illnesses globally each year, after *Campylobacter*. Furthermore, non-typhoidal *Salmonella enterica* predominates in deaths caused by foodborne diarrheal disease agents worldwide [4]. These results indicate that *Salmonella* infections contribute significantly to the global burden of foodborne illnesses. The genus *Salmonella* can be divided into the following two species: *Salmonella bongori* and *Salmonella enterica*. The former is rarely associated with human infections. The latter includes the following six subspecies: *S. enterica* subsp. *enterica* (I), *S. enterica* subsp. *salamae* (II), *S. enterica* subsp. *arizonae* (IIIa), *S. enterica* subsp. *diarizonae* (IIIb), *S. enterica* subsp. *houtenae* (IV), and *S. enterica* subsp. *indica* (VI) [5]. Within these subspecies, strains can be further classified into serotypes, and more than 2600 serotypes have been described based on combinations of different somatic (O) and flagellar (H) antigens using the Kauffman–White scheme [6]. More than half of these serotypes belong to *S. enterica* subsp. *enterica*, which is the main cause of gastroenteritis in warm-blooded animals [7]. Other *Salmonella* subspecies (II–VI) and *S. bongori* are associated with diseases in cold-blooded organisms and occasionally cause systemic diseases in humans. Different *Salmonella* serotypes exhibit different genetic characteristics, host specificity, pathogenicity, and antimicrobial resistance, leading to the development of distinct epidemiological and clinical symptoms [8,9].

The pathogenicity of *Salmonella* is associated with multiple virulence factors and antimicrobial resistance [10]. The study of antimicrobial resistance and virulence genes of foodborne pathogens is very common in recent years [11,12,13,14]. *Salmonella* uses multiple virulence determinants such as flagella, capsules, plasmids, adhesion systems, and the type 3 secretion system (T3SS) encoded by *Salmonella* pathogenicity island (SPI) to colonize the host by attaching, invading, and bypassing the gastrointestinal defense mechanisms of the hosts [9]. The SPI is an unstable chromosomally located DNA segment containing virulence-related genes. To date, 24 SPIs have been recognized [15]. Of these, the virulence genes involved in the intestinal phase of infection were located in SPI-1 and SPI-2; the remaining SPIs are involved in physiological processes, such as *Salmonella* survival in host cells, magnesium and iron uptake, multidrug resistance (MDR), and the development of systemic infections [16,17]. In addition to SPI, *Salmonella* virulence-related genes are distributed in virulence plasmids, bacteriophages, flagella, and enterotoxins, which play various physiological roles in the host [18]. Understanding *Salmonella* virulence islands and the composition of virulence genes is essential for predicting their pathogenic potential. Recently, *Salmonella* resistance has become an increasing problem due to the misuse of antibiotics in poultry farming and clinical medicine. A recent study has reported that non-typhoidal *Salmonella* is resistant to almost all classes of antibiotics, which significantly limits the choice of effective drugs and poses a considerable challenge in the treatment of salmonellosis [19]. Mobile elements, such as plasmids and integrons, play an important role in *Salmonella* antimicrobial resistance because they mediate the horizontal transfer of antimicrobial resistance genes (ARGs) to exacerbate the spread of resistance [20]. Ongoing research on *Salmonella* resistance can guide drug selection and inform the development of new drugs.

A previous study has reported that *Salmonella* can be transmitted across geographic regions through contaminated food and can form independent evolutionary branches in different regions, thereby increasing the genetic diversity of *Salmonella* populations [21]. Therefore, studying the genetic relationships among *Salmonella* species is very important. Several molecular typing methods have been used in epidemiological studies of *Salmonella* to determine the genetic relationships between isolates, e.g., pulsed-field gel electrophoresis, enterobacterial repetitive intergenic consensus polymerase chain reaction, and multiple-locus variable number tandem repeat analysis [10]. Currently, whole-genome sequencing (WGS) is an accurate tool for predicting the genetic characterization of bacterial strains as well as their potential pathogenicity and antimicrobial resistance [22]. WGS can also provide insights into the genomes of pathogens, including information on species, serotypes, resistance and virulence factors, pathogenicity mechanisms, and their evolution in pathogens [23,24].

Wenzhou is an important commercial city on the southeastern coast of China, located near Shanghai, with frequent import and export trade. An in-depth understanding of the molecular characteristics of *Salmonella* and an assessment of the risk of importation can be effective in preventing and controlling infections with this pathogen. In this study, we analyzed 24 *Salmonella* strains isolated from different sources (human stool, poultry, and food) using WGS. The main objective was to study the ARGs, virulence factors, and genetic diversity of these strains. By analyzing the molecular characteristics of the strains, we were able to assess their drug resistance and pathogenic potential. These results may serve as a reference for the prevention and control of bacterial diseases.

## 2. Methods

### 2.1. Salmonella Isolation and DNA Extraction

A total of 24 *Salmonella* strains recovered from a variety of foods (raw poultry and flour products) and clinical samples (stool) of patients associated with diarrhea in Wenzhou in 2020 were included in the study (Wenzhou Center for Disease Control and Prevention Biobank), as shown in Table 1. Sterile containers were used during sampling, and the samples were transported to the Wenzhou CDC following aseptic procedures. Aseptic containers were used for sampling and the collected samples were transported to the laboratory within four hours under low temperature storage conditions in accordance with aseptic procedures. All samples were tested with reference to GB 4789.4-2016 China National Standard for Food Safety Food Microbiology Test for *Salmonella* Test. Firstly, 2–3 suspicious colonies were picked from *Salmonella* chromogenic medium, inoculated in Triple Sugar Iron Agar Medium, and incubated at 36 °C ± 1 °C for 18~24 h. The test phenomenon was observed, and the red color of the slant surface and the blackening of the bottom layer were judged to be suspicious *Salmonella* spp. Suspect colonies were picked and inoculated on Tryptone Soy Agar (TSA) plates, then systematically biochemically identified using GN colorimetric identification cards (Bio Mérieux China, Shanghai, China) and double-checked for identification using the microbial mass spectrometry identification system MALDI Biotyper Smart (Bruker Beijing Scientific Technology Co. Ltd., Beijing, China). Amongst 18 strains isolated from raw poultry (S1–S3, S5–S13, and S19–S24), five strains were isolated from human stool (S14–S18) and one strain was isolated from flour products (Table 1). Of these, human stool samples were obtained from patients with foodborne illness diarrhea. Strains were centrifuged at 6010× *g* for 1 min (Allegra 64R Benchtop Centrifuge, Beckman Coulter, Pasadena, US), pellets with genomic DNA was extracted from *Salmonella* using a bacterial genomic DNA extraction kit GK1072 (Shanghai Jereh Bioengineering Co., Ltd., Shanghai, China) and WGS was performed.

### 2.2. WGS, Assembly and Genome Annotation

*Salmonella* genomes were sequenced on the Illumina NovaSeq platform using 2 × 150 bp paired ends according to the manufacturer’s protocols (Illumina TruSeq Nano DNA LT, San Diego, US) at a commercial company (Shanghai Personal Biotechnology Co., Ltd., Shanghai, China). The Illumina TruSeq DNA Sample Preparation Guide was used to construct a genomic sequencing library. After the libraries were qualified, they were sequenced using Illumina NovaSeq according to the effective concentration. The results were stored in a paired-end FASTQ format, which contained sequence information of sequencing reads and corresponding sequencing quality information. To ensure the quality of the subsequent information analysis, AdapterRemoval v2.2.2 was used to remove adapter contamination from the downlinked data. Illumina reads were assembled de novo using SPAdes v3.12.0 to obtain genome sequence contigs. This step was bp corrected using Pilon v1.18. Genome annotation using prokka v1.14.6.

### 2.3. Bioinformatics Analyses

For further genome analysis, raw reads were submitted to EnteroBase [25]. In EnteroBase we called the Achtman 7 Gene MLST scheme for multilocus sequence typing (MLST). Seven *Salmonella* housekeeping loci (*aroC*, *dnaN*, *hemD*, *hisD*, *purE*, *sucA*, and *thrA*) were used for this analysis and were also used to assign numbers to sequence types [25]. In silico serotyping was performed using the SeqSero v1.2 tool (https://cge.food.dtu.dk/services/SeqSero/) [26]. We also used the EnteroBase and cgMLST schemes to construct a phylogenetic tree. In total, 3002 loci were used for cgMLST analysis. A Grape Tree was constructed using the neighbor-joining (NJ) algorithm and visualized using iTOL v5.1.1 (https://itol.embl.de/).

Detection of acquired ARGs and chromosomal point mutations in the genome was performed using ResFinder v4.1 (https://cge.food.dtu.dk/services/ResFinder/) with a minimum threshold of 90% identity and 60% coverage [27]. PlasmidFinder v2.0.1 was used to identify plasmids in the genome (https://cge.food.dtu.dk/services/PlasmidFinder/) with a minimum threshold of 95% identity and 60% coverage [28]. Integron Finder v2.0.1 was used, followed by BLASTp analysis to identify the I integron in the genome data [29]. Genomes were annotated for virulence factors using the Virulence Factor Database (VFDB) Online Annotation tool VFanalyzer (http://www.mgc.ac.cn/VFs/) [30]. SPIFinder was performed using the local command line version of SPIFinder of the Center for Genomic Epidemiology for *Salmonella* available at (https://cge.food.dtu.dk/services/SPIFinder/) with a minimum threshold of 95% identity and 60% coverage [31].

### 2.4. Data Availability

Sequencing data for all *Salmonella* strains were deposited in NCBI under Bioproject PRJNA831306. Accession numbers were JALPKT000000000–JALPLQ000000000 (Table 1).

The 314 *S*. Liverpool (Table S3) and 13 *S*. Typhimurium (Table S4) strains used for cgMLST analyses, respectively, were obtained from the EnteroBase database (https://enterobase.warwick.ac.uk/species/index/senterica). The 314 *S*. Liverpool from S14–S18 and its closely related strains. A total of 13 *S*. Typhimurium strains were from S11 and its closely related strains. Sequencing primer information is provided in Table S5.

## 3. Results

### 3.1. Genomic Features of the Salmonella Strains

After quality control filtering, the percentage of bases with identification accuracies above 99% (Q20) and 99.9% (Q30) exceeded 97% and 92%, respectively, the N50 ranged from 73,840 to 534,429, and the depth of coverage ranged from 193 to 309x, which demonstrated that the reliability of the sequencing data was high. A total of 1713 contigs were generated by assembling, and each genome contained contigs varying from 32 to 240. Among the 24 strains, genome sizes were roughly equal and ranged from 4.58 to 5.08 Mb with a GC content of 51.9–52.3%. Functional annotation predicated that that these strains had 4393–4405 CDSs, 72–74 tRNA, and 2–4 rRNAs (Table S1).

### 3.2. The Serotyping of Salmonella Isolates Using MLST Typing

Among the 24 *Salmonella* isolates, 10 serotypes were identified. *S*. Typhimurium was the most prevalent serotype (*n* = 7, 29.2%), followed by Liverpool (*n* = 5, 20.8%), London (*n* = 4, 16.7%), and Goldcoast (*n* = 2, 8.3%). Only one isolate was present for each of the following serotypes: Javiana, Kentucky, Corvallis, Meleagridis, Infantis, and Anatum (Table 1).

Based on the available data in the *S. enterica* MLST database, 12 distinct sequence types were recognized; three different sequence types were identified as ST19, ST34, and ST1544 in *S.* Typhimurium, and the other serotypes corresponded to a unique sequence type. The sequence types included ST155, ST19, ST34, ST1544, ST358, ST1959, ST463, ST1541, ST198, ST64, ST32, and ST9074 (Table 1).

### 3.3. Identification of ARGs

**Figure 1:**
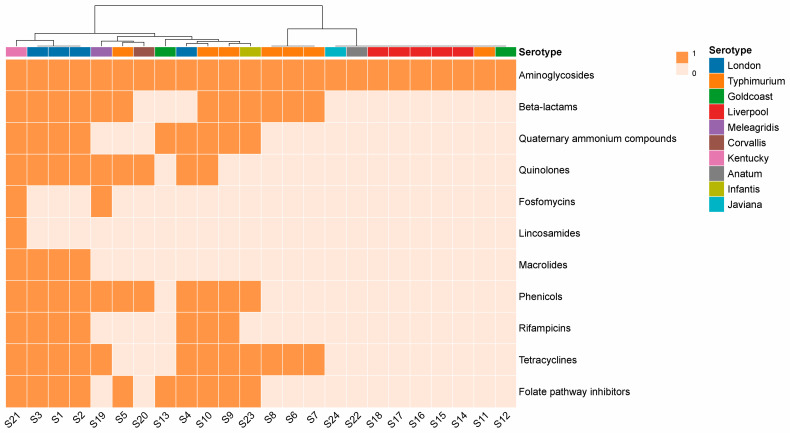
The frequency of ARGs in different drug classes were described. ARGs of 24 *Salmonella* isolated from Wenzhou are obtained using ResFinder. The shades of color in the legend represent the presence (1) and absence (0) of that class of gene.

A total of 11 classes of ARGs were detected in 24 strains. Aminoglycoside resistance genes showed the highest frequency (100%, 24/24), followed by beta-lactams (50%, 12/24), tetracyclines (50%, 12/24), phenicols (45.83%, 11/24), folate pathway inhibitors (41.67%, 10/24), quinolones (37.5%, 9/24); quaternary ammonium compounds (37.5%, 9/24), rifampicins (29.17%, 7/24), macrolides (16.67%, 4/24), fosfomycins (8.33%, 2/24), and lincosamides (4.17%, 1/24) (Figure 1). There were differences in the types of resistance genes carried by different serotypes. All isolates contained at least one type of ARG, of which 15 contained more than 3 types of ARGs and 10 contained more than 5 types of ARGs. Aminoglycoside resistance genes show diversity, including *aac(3)-Id*, *aac(3)-IId*, *aac(3)-IV*, *aac(6’)-Iaa, aadA1*, *aadA16*, *aadA17*, *aadA2*, *aadA7*, *aph(3’)-Ia*, *aph(3’’)-Ib*, *aph(4)-Ia*, *aph(6)-Id*, *rmtB*, and *aac(6’)-Ib-cr*. Five isolates carried the quinolone-modifying enzyme gene *aac(6’)-Ib-cr*. In total, 12 isolates carried beta-lactams genes, all belonging to extended-spectrum-beta-lactamase (ESBL) genotypes, including TEM, CTX-M, and OXA genotypes. One *S.* Kentucky strain had TEM combined with the CTX-M dual genotype, and one *S.* Typhimurium strain had TEM combined with the OXA dual genotype. Six isolates carried both *qnr* and ESBL genes. *S.* Kentucky carried up to 11 classes of ARGs. Lnu(F) and *fosA3* genes were found only in *S*. Kentucky. FosA7 was detected in one *S*. Meleagridis strain (Table 1).

In total, 24 point mutations were detected in *gyr*A and *par*C associated with quinolone resistance in 24 *salmonella* strains. No mutations were detected in *gyr*B or *par*E. The most common mutation in *gyr*A was S83Y, followed by S83F, D87N, and D87Y. The most common mutation in *par*C was T57S, followed by S80I. Among the twenty isolates with point mutations detected, eight strains carried plasmid-mediated *qnr* genes, and four strains of *S*. London carried both *qnr* and *aac(6’)-Ib-cr* genes (Table 1), suggesting that there is an extended spectrum of resistance to quinolones. A strain of *S*. Kentucky with four point mutations also carried the *qnrS1* gene.

### 3.4. Plasmid and I Integron

In total, 29 bacterial plasmids were identified in 24 strains. S9 contains the Folate pathway inhibitor resistance gene *sul2* and aminoglycoside resistance genes *aph(6)-Id* and *aph(3’’)-Ib*. Ten isolates did not carry any plasmids, fourteen isolates had at least one plasmid, and nine isolates carried two or more plasmid types. IncFIB was the dominant incompatibility group (Table 2).

Five complete class I integrons have been identified. All four strains of *S*. London contain one I integron carrying the same gene cassette arrays *aadA16-dfrA27-ARR-3-aac(6’)-Ib-cr*. A strain of *S*. Infantis was found to have one I integron carrying the gene cassette *aadA1*. A CALIN-type integron in the form of a gene cassette was found in one *S*. Typhimurium, carrying the gene cassette *aac(6’)-Ib-cr—blaOXA-1-catB3—ARR-3* (Table 2).

### 3.5. Virulence Genes

The results of the online annotation of virulence factors for the 24 isolates using the virulence factor database VFBD are shown in Table S2. Some genes (95/234) were conserved among all the isolates. The phage-associated gene *sodCI*, prophage-encoded gene *gogB*, macrophage-inducible gene *mig-5*, plasmid-mediated genes *spvB*, *spvD*, *rck*, and plasmid-encoded fimbrial gene *sefABCD* were detected only in *S*. Typhimurium. In particular, *S*. Typhimurium, numbered S9, lacked all virulence plasmid-associated genes. *S*. Liverpool lacked the multiple fimbrial operons *stf*, *sti*, *stj*, *stk*, *lpf*, *peg*, and *tcf*, which may affect the ability of bacteria to invade and colonize. *S*. Liverpool also lacked the bacterial effector *avrA*. Additionally, the cytolethal-distending toxin *cdtB* was detected in one *S*. Javanica and two *S*. Goldcoast. The yersiniabactin (*ybt)* gene was detected in a strain of *S*. Infantis (Table S2).

### 3.6. The SPI Profiles

**Figure 2:**
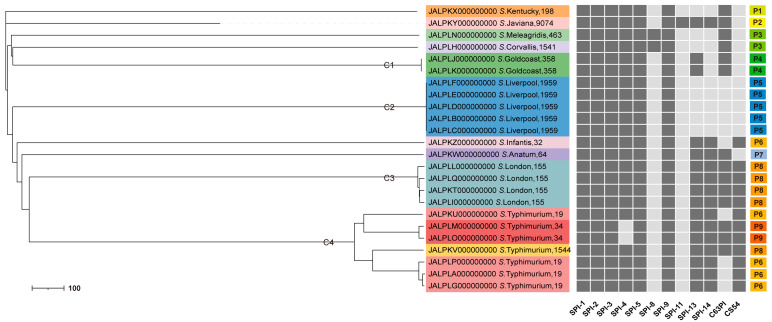
cgMLST tree and SPI profiles of 24 *Salmonella* isolates in this study. cgMLST tree using the 3002 locus cgMLST scheme provided by EnteroBase. Assignment of serotypes into four main clusters contained the main serotypes detected; *S*. Typhimurium, Liverpool, London, and Goldcoast, respectively, defined as C1, C2, C3, and C4. SPI profiles were obtained using the SPIFinder. Varied according to cgMLST clusters, nine SPI profiles were identified, named P1 to P9. The scale bars represent paired allelic differences at the cgMLST locus.

All 24 *Salmonella* isolates carried the same five SPIs, namely SPI-1, SPI-2, SPI-3, SPI-5, and SPI-9. SPI-4 was detected in all isolates except for two strains of *S*. Typhimurium of the ST34 genotype. SPI-8 was detected in only one strain of *S*. Corvallis and one strain of *S*. Meleagridis. SPI-11 was detected in only one strain of *S*. Javiana. Nine SPI profiles were identified, named P1 to P9, and most ST strains corresponded to the same SPI profiles. One strain of *S*. Corvallis and one strain of *S*. Meleagridis with similar phylogenetic affinities shared the same SPI profile. A strain with genotype ST1544 in *S*. Typhimurium had the same SPI profile as *S*. London. Additionally, the three sequence types of *S*. Typhimurium, ST19, ST34, and ST1544, corresponded to three SPI profiles, P6, P9, and P8, respectively (Figure 2).

### 3.7. Phylogenetic Relationship of Salmonella Strains by cgMLST Analysis

The assignment of serotypes to the four main clusters was consistent with the global phylogenetic tree. All the remaining serotypes were unique. Four clusters contained the main serotypes detected, namely *S*. Typhimurium, Liverpool, London, and Goldcoast, respectively, defined as C1, C2, C3, and C4 (main branches separated by nodes with a well-defined genetic distance). Two sub-clusters within the cluster C4 (Figure 2) provide evidence of their genetic diversity.

**Figure 3:**
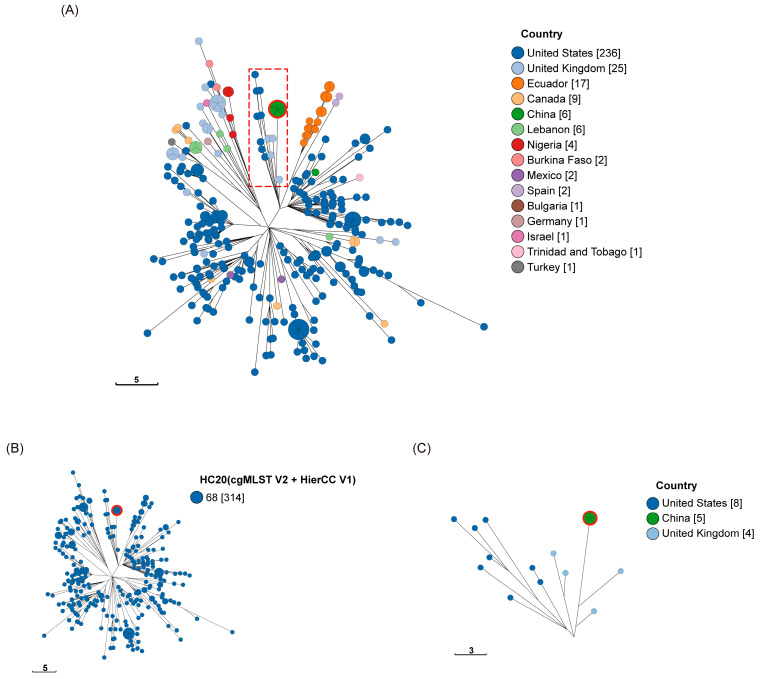
Phylogenetic relationships among *S*. Liverpool (ST1959) by cgMLST analysis based on country of origin (**A**) and HC20 (68) of HierCC (**B**). Minimum spanning tree of 314 *S.* Liverpool strains was generated from cgMLST + HierCC data using the 3002 locus cgMLST scheme provided by EnteroBase. The isolates within this analysis were from S14 to S18 (position of the red circle in the picture) and its closely related strains. (**A**): The colors distinguish the countries of origin of the strains, with the majority coming from the United States (US), followed by the United Kingdom (UK). The red rectangular dashed boxes indicate the clustering of the five *S*. Liverpool strains (S14–S18) in our study with strains from the UK and the US. (**B**): All strains were obtained by screening for the HC20 (68) cutoff value. Thus, all strains differed by no more than 20 alleles. (**C**): A magnified view of the red rectangular dashed boxes in Figure (**A**). In the magnified view, they appear to be closer to the UK strains. The scale bars represent paired allelic differences at the cgMLST locus.

Based on EnteroBase cgMLST with Hierarchical Clustering (HierCC), *S*. Liverpool in our study appeared to be related to strains prevalent in the United Kingdom (UK) and the United States (US). They were found simultaneously in both countries, and it is speculated that they may share a common ancestor. *S*. Liverpool in our study appeared to be more closely related to several strains in the UK (see Figure 3 and Table S3).

**Figure 4:**
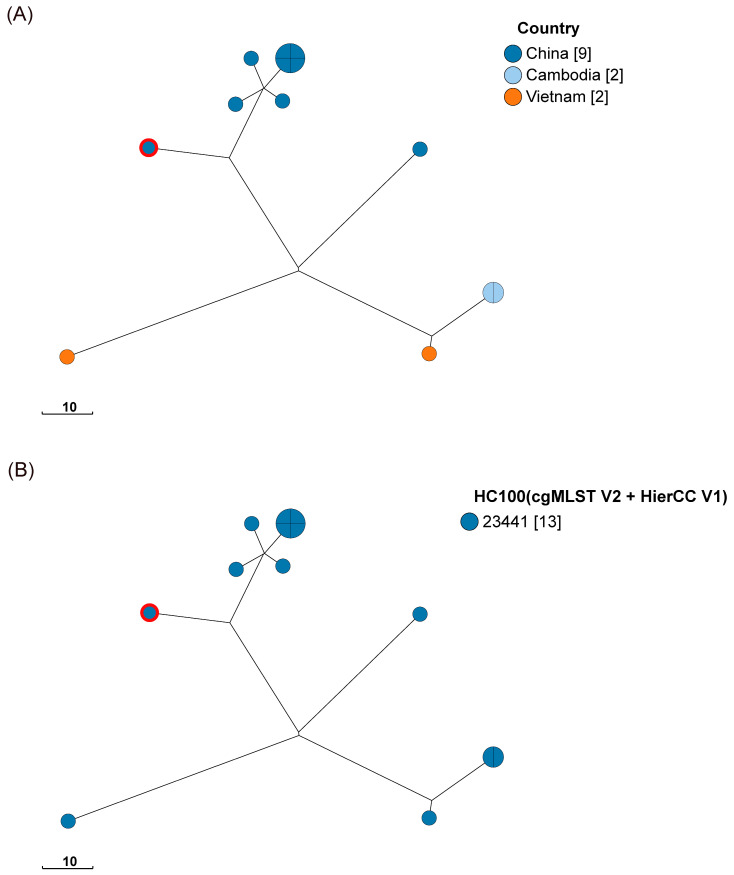
Phylogenetic relationships among the *S.* Typhimurium (ST1544) by cgMLST analysis based on country of origin (**A**) and HC100 (23441) of HierCC (**B**). Minimum spanning tree of 13 *S.* Typhimurium strains was generated from cgMLST + HierCC data using the 3002 locus cgMLST scheme provided by EnteroBase. The isolates within this analysis were from S11 (position of the red circle in the picture) and its closely related strains. (**A**): The colors distinguish the countries of origin of the strains: China, dark blue; Cambodia, light blue; Vietnam, orange. (**B**): All strains were obtained by screening for the HC100 (23441) cutoff value. Thus, all strains differed by no more than 100 alleles, suggesting that ST1544 in our study was associated with the prevalence of these strains. The scale bars represent paired allelic differences at the cgMLST locus.

In addition, based on the cgMLST with HierCC phylogenetic tree, the ST1544 strain in our study from raw poultry was associated with the prevalence of five strains isolated from wild animals in Yangzhou, China, two strains from human and food samples in Vietnam, and two strains from environmental samples in Cambodia (Figure 4 and Table S4).

## 4. Discussion

In this study, a collection of 24 *S. enterica* strains was studied through WGS and subsequent bioinformatics analysis to determine the distribution of serotypes, sequence types, virulence genes, ARGs, plasmid sequences, integron sequences, and SPIs.

### 4.1. Serotyping and MLST

Relevant studies have confirmed the high accuracy of WGS-based serotyping [32]. It has been widely accepted as a promising tool for the high-resolution typing of enteric pathogens [33]. It is gradually replacing the traditional methods of foodborne pathogen typing [34,35]. Our study identified the presence of *S*. Liverpool based on WGS data. However, the presence of *S*. Liverpool has not been reported in previous studies in Wenzhou [36,37,38]. Based on EnteroBase cgMLST with Hierarchical Clustering (HierCC), *S*. Liverpool in our study appeared to be related to strains prevalent in the UK and US. In Figure 3, S14–S18 are observed to be more closely related to several strains in the UK, suggesting that they may share a common origin (see Figure 3 and Table S3). As *Salmonella* can be transmitted through contaminated food in different regions, the risk of spreading the pathogen has increased in recent years with the increase in global trade [21,39]. The establishment of an international surveillance network would be beneficial in controlling the risk of international transmission of *Salmonella*.

The results of MLST of *Salmonella* strains based on seven housekeeping genes showed that none of the serotypes had multiple MLST types, except for *S*. Typhimurium. ST19 and ST34 are the two most common sequence types reported in multinational studies [40,41,42]. In contrast, ST1544 has been reported in only a few studies.

Our study reports for the first time the occurrence of ST1544 in the Wenzhou region, increasing the population diversity of *S*. Typhimurium in the region. Owing to extensive host contamination, its genetic characteristics for contaminating different hosts are unknown. In the future, it may develop into a novel contaminant in Southeast Asia and pose a threat to the coastal cities in eastern China.

### 4.2. Antimicrobial Resistance Determinants

ARG analysis showed that these strains may be resistant to multiple antibiotics. The high frequency of detection of aminoglycosides, tetracyclines, beta-lactams, phenicols, and folate pathway inhibitor ARGs in the isolates from poultry samples may be related to the fact that amoxicillin, florfenicol, ciprofloxacin, sulfamethoxazole, and streptomycin are widely used for the prevention or treatment of bacterial diseases in most of the poultry farms in China. The proportion of ESBL-producing strains reached 50%, which was significantly higher than that reported by Chen Sucai et al. in Wenzhou [43]. Notably, ESBLs can hydrolyze a variety of antibiotics such as penicillin, cephalosporins, and aztreonam. Secondly, its presence is more likely to lead to cross-resistance in bacteria [44,45], probably because the plasmids encoding ESBLs also carry resistance genes for quinolones, aminoglycosides, and other antibiotics [46]. However, all ESBL-producing strains in our study carried aminoglycoside, quinolone, tetracycline, and chloramphenicol resistant genes, with potential MDR. A previous study showed that when CTX-M-55 was combined with TEM, it significantly reduced susceptibility toward piperacillin–tazobactam and cefotetan. When CTX-M-55, TEM, and SHV genes were present together, the strains were 100% resistant to both antibiotics [47]. This suggests that strains containing dual ESBL genotypes or above can significantly decrease antibiotic susceptibility and broaden the spectrum of antimicrobial resistance. A strain of *S*. Kentucky and a strain of *S*. Typhimurium containing dual ESBL genotypes were observed in our study, and the resistance of the strains to cephalosporins and beta-lactam combinations is of concern.

The high level of resistance to quinolones in strains can be attributed to the accumulation of point mutations in genes encoding cellular topoisomerases and the acquisition of several auxiliary mechanisms, such as efflux-pump-encoding *qepA* and *oqxAB*, *Qnr* proteins, and the aminoglycoside acetyltransferase *aac(6′)-Ib-cr*, which increase the level of expressed resistance [48]. Although these plasmid-mediated quinolone resistance determinants confer low levels of resistance toward quinolones, they can assist in the emergence of other chromosomally encoded quinolone resistance mechanisms [49]. The aminoglycoside acetyltransferase *aac(6’)-Ib-cr* is a variant of *aac(6’)-Ib* that induces resistance toward aminoglycosides and fluoroquinolones. The remaining three appeared at high frequencies in our study, except for the efflux pump. However, the frequency of point mutations was as high as 83% (20/24), and *S*. London also contained *aac(6’)-Ib-cr* and *qnrB6*. *S*. Kentucky had the most point mutations and contained *qnrS1*, suggesting that the *S*. London and *S*. Kentucky in this study had a strong potential for resistance to quinolones. We recommend the rational use of quinolones in poultry farming and clinical practice to prevent the acceleration of this resistance mechanism in the future. Furthermore, for the treatment of diseases caused by ciprofloxacin-resistant *Salmonella*, a third-generation cephalosporin, such as cefotaxime or ceftriaxone, is preferred [50]. Second, ciprofloxacin-resistant strains were predicted to carry ESBL at a frequency of 50% (11/22) in our study. This means that the scope of clinical drugs is further decreased, and new drugs may need to be developed to address this situation in the future. 

In addition to ARGs, mobile genetic elements such as plasmids and integrons play a key role in the spread and persistence of antimicrobial resistance [51]. IncFIB (also known as the ColV plasmid) was the most common plasmid type in our study and may be related to virulence plasmids. A previous study found that when *S*. Kentucky acquired the IncFIB plasmid, it increased its ability to colonize the chicken cecum and caused significant extra-intestinal disease [52]. Here, IncFIB plasmids did not carry resistance genes, which may be due to the lack of such genes in virulence plasmids or the inability of the draft genome to assemble complete plasmid sequences [28]. Although most plasmids do not encode known ARGs, they can bind to other transposable elements to form MDR clusters, facilitating their spread [20].

Integrons can trap exogenous resistance genes, causing the spread of resistance genes among bacterial populations. Our results showed that 100% of *S*. London contain a complete class 1 integron regardless of the source. Each integron carries the same gene cassette arrays *aadA16-dfrA27-ARR-3-aac(6’)-Ib-cr*, which is associated with resistance toward aminoglycosides, folate pathway inhibitors, rifampicins, and quinolones. This indicates that the contribution of integrons to MDR in *S*. London is significant; therefore, the spread of resistance at the genetic level in *S*. London is concerning. Second, the quinolone-modifying enzyme genes *aac(6’)-Ib-cr* detected in five samples were all located on the integrons, suggesting that the integron is highly capable of capturing and propagating the *aac(6’)-Ib-cr* gene.

However, the detection of ARGs does not necessarily mean that the strain is resistant to these antimicrobials. There may be other factors such as mutation and genetic integrity. For example, the aac(6’)-Iaa gene, which is common in *Salmonella*, has been reported to be a cryptic gene that requires mutation to activate it [53]. In the current study, the *S.* Liverpool strains only presented this ARG, and it is possible that they do not phenotypically exhibit this resistance. Further studies are needed in the future to confirm such a possibility. Phenotypic testing is needed to further validate this possibility. As a limitation of the study, in the future we will pay more attention to gene and phenotype association analysis.

### 4.3. Virulence Determinants

The pathogenicity of *Salmonella* is closely associated with its virulence genes. A cluster of relatively concentrated virulence genes constitutes the SPI. At least 24 SPIs have been identified in the genus *Salmonella*, which plays a crucial role in the pathogenesis of the strains [54]. SPI-1 to SPI-5 were highly conserved. SPI-1 and SPI-2 were the two main virulence determinants of *S. enterica* and encoded the T3SS. SPI-1 is required for host cell invasion and the induction of macrophage apoptosis; SPI-2 is required for *Salmonella* survival within macrophages and causes systemic infection; SPI-3 and SPI-4 are both associated with intracellular survival; and SPI-5 is associated with host cell invasion and inflammatory diseases. Other SPIs are less well studied than SPI-1 to SPI-5 but may also have specific functions, such as invasion and colonization [55,56]. SPI-9 in *Salmonella* Typhi contributes to epithelial cell adhesion [57]. SPI-11 and SPI-13 are involved in intracellular survival and promote systemic infection in mice [58]. SPI-14 is a key virulence factor for systemic infections in chickens [59]. C63PI is an iron transport system in SPI-1 [60]. The functions of SPI-8 and CS54 need to be further investigated. In our study, the investigation of 24 strains showed that five SPIs, namely SPI-1, SPI-2, SPI-3, SPI-5, and SPI-9, were conserved in all strains. A previous study showed the prevalence of SPI-1 to SPI-5, SPI-13, and SPI-14 and the absence of SPI-7, SPI-8, and SPI-15 in all non-*Salmonella* typhi isolates. Nevertheless, SPI-8 was detected in one strain of *S*. Meleagridis and one strain of *S*. Corvallis in our study. To the best of our knowledge, this is the first study to report SPI-8 in these two serotypes.

Our research showed that the SPI profiles (P1–P9) varied according to the cgMLST clusters (Figure 2). For example, the dominant SPI profiles, P4, P5, and P8, were represented among clusters carrying *S*. Goldcoast (ST358), Liverpool (ST1959), and London (ST155), respectively. More importantly, the three STs in *S*. Typhimurium corresponded to three SPI profiles, suggesting that SPI profiles are likely to influence the classification of *Salmonella* genotypes. Furthermore, a strain of *S*. Corvallis and a strain of *S*. Meleagridis with similar phylogenetic relationships shared the same SPI profiles, indicating that serotypes with close genetic relationships may have the same distribution of virulence factors. The more closely related *S*. London and *S*. Typhimurium possessed broader SPI profiles, suggesting that they may have greater pathogenic potential.

Fimbriae are the most common adhesion systems that play a major role in the pathogenesis of *Salmonella*. It has been shown that fimbriae represent a source of diversity among *Salmonella* serotypes, which is differentially expressed across serotypes and found in specific patterns [61]. In our study, the fimbrial operons *bcf*, *fim*, *inv*, *csg*, *stb*, and *sth* were present in all isolates. These genes may be part of a core gene that is key for *Salmonella* to invade the host and cause infection. Other operons *lpf*, *peg*, *saf*, *ste*, *stf*, and *sti* exist depending on serotypes (Table S2). Virulence plasmids of *Salmonella* are important for systemic infection in animal models. *Spv* genes may accelerate *Salmonella* growth in host cells and affect the interactions between *Salmonella* and the host immune system [62]. In our study, the *spv* gene was found only in *S*. Typhimurium, which may also be responsible for making it more pathogenic. Moreover, the phage-associated gene *sodCI*, prophage-encoding gene *gogB*, macrophage-inducible gene *mig-5*, plasmid-mediated gene *rck*, and plasmid-encoded fimbrial gene *sefABCD* were only detected in *S*. Typhimurium. These results indicate that *S*. Typhimurium is the most virulent strain of *Salmonella* causing poultry infections and threatening food safety in Wenzhou, China.

### 4.4. Genetic Diversity of the Salmonella Isolates

In our study, genetic relationships between the strains were constructed using cgMLST on the EnteroBase platform. The construction of phylogenetic clusters of isolates was based on 3002 core-genome loci. This clustering by serotype was also presented in the study of Hassena et al. [22]. Due to the dispersion of serotypes, more detailed clustering could not be observed. However, the sub-clustering of *S*. Typhimurium indicated that it has richer genetic diversity in the region.

The cgMLST scheme uses a consistent set of conserved loci and allele assignments with the advantage of being easily and consistently applied across laboratories and jurisdictions. In addition, EnteroBase supports HierCC, a new approach that supports the analyses of population structures based on cgMLST at multiple-level resolutions. EnteroBase reported the most reliable *Salmonella*-specific subset of the HierCC clusters [25]. In our study, *S*. Liverpool found in the Wenzhou area, appears to be closely related to several strains in the UK (HC20), suggesting that they may share a common origin (Figure 3). The discovery of this important information would be beneficial for monitoring *Salmonella* in Wenzhou.

## 5. Conclusions

In this study, we explored the association between *Salmonella* serotypes and antimicrobial resistance, genotypes and, pathogenic potential and confirmed the presence of an imported risk. *Salmonella* contains a variety of ARGs, SPIs, virulence plasmids, multidrug resistance plasmids, phages, and integrons that influence its classification of *Salmonella* and shed light on the causes of the severity of this bacterial disease. Hence, obtaining the genome sequence of *Salmonella* will not only help to improve the reproducibility and accessibility of genomic analyses, but also contribute to future surveillance and epidemiological investigations of salmonellosis. The data obtained in this study can provide reference for the prevention and control of bacterial diseases and dynamic monitoring.

## Figures and Tables

**Table 1:** Sources, serotype and antimicrobial resistance genes (ARGs) of *Salmonella* (Location * Wenzhou, Year * 2020, Species * *S. enterica*).

Sample	Genome Accession	Source	Serotype	ST	ARGs	QRDR	
						GyrA	ParC
S1	JALPKT000000000	raw poultry	London	155	*aac(3)-Iid* *, aac(6’)-Iaa* *, aac(6’)-Ib-cr, aadA16* *, aph(3’’)-Ib* *, aph(6)-Id, blaTEM-1B, qnrB6, tet(A), sul1, sul2, catA2, floR, ARR-3, dfrA27, qacE, mph(A)*	-	*T57S*
S2	JALPLI000000000	raw poultry	London	155	*aac(3)-Iid, aac(6’)-Iaa, aac(6’)-Ib-cr*, *aadA16, aph(3’’)-Ib, aph(6)-Id, blaTEM-1B, qnrB6, tet(A), sul1, sul2, floR, ARR-3, dfrA27, qacE, mph(A)*	-	*T57S*
S3	JALPLL000000000	raw poultry	London	155	*aac(3)-Iid, aac(6’)-Iaa, aac(6’)-Ib-cr*, *aadA16, aph(3’’)-Ib, aph(6)-Id, blaTEM-1B, qnrB6, tet(A), sul1, sul2, floR, ARR-3, dfrA27, qacE, mph(A)*	-	*T57S*
S4	JALPLQ000000000	food	London	155	*aac(6’)-Iaa, aac(6’)-Ib-cr, aadA16, aph(3’’)-Ib, aph(6)-Id, qnrB6, tet(A), sul1, sul2, floR, ARR-3, dfrA27, qacE*	-	*T57S*
S5	JALPKU000000000	raw poultry	Typhimurium	19	*aac(6’)-Iaa, blaCTX-M-65, OqxA, OqxB, qnrS2, sul1, floR*	-	-
S6	JALPLA000000000	raw poultry	Typhimurium	19	*aac(6’)-Iaa, aph(3’’)-Ib, aph(6)-Id, blaTEM-1B, tet(A)*	*S83Y*	-
S7	JALPLG000000000	raw poultry	Typhimurium	19	*aac(6’)-Iaa, aph(3’’)-Ib, aph(6)-Id, blaTEM-1B, tet(A)*	*S83Y*	-
S8	JALPLP000000000	raw poultry	Typhimurium	19	*aac(6’)-Iaa, aph(3’’)-Ib, aph(6)-Id, blaTEM-1B, tet(A)*	*S83Y*	-
S9	JALPLM000000000	raw poultry	Typhimurium	34	*aac(3)-Iid, aac(6’)-Iaa, aph(3’’)-Ib, aph(6)-Id, blaTEM-1B, tet(B), sul1, sul2, floR, ARR-3, dfrA27, qacE*	-	-
S10	JALPLO000000000	raw poultry	Typhimurium	34	*aac(3)-IV, aac(6’)-Iaa, aac(6’)-Ib-cr, aph(3’’)-Ib, aph(4)-Ia, aph(6)-Id, blaOXA-1, blaTEM-1B, tet(B), sul1, sul2, catB3, ARR-3, qacE*	-	-
S11	JALPKV000000000	raw poultry	Typhimurium	1544	*aac(6’)-Iaa*	-	-
S12	JALPLJ000000000	raw poultry	Goldcoast	358	*aac(6’)-Iaa*	-	*T57S*
S13	JALPLK000000000	raw poultry	Goldcoast	358	*aac(6’)-Iaa, aac(3)-Iid, sul1, qacE, fosA7*	-	*T57S*
S14	JALPLB000000000	human stool	Liverpool	1959	*aac(6’)-Iaa*	-	*T57S*
S15	JALPLC000000000	human stool	Liverpool	1959	*aac(6’)-Iaa*	-	*T57S*
S16	JALPLD000000000	human stool	Liverpool	1959	*aac(6’)-Iaa*	-	*T57S*
S17	JALPLE000000000	human stool	Liverpool	1959	*aac(6’)-Iaa*	-	*T57S*
S18	JALPLF000000000	human stool	Liverpool	1959	*aac(6’)-Iaa*	-	*T57S*
S19	JALPLN000000000	raw poultry	Meleagridis	463	*aac(6’)-Iaa, aph(3’)-Ia, blaTEM-1A, OqxA, OqxB, qnrS1, tet(A), floR*	-	*T57S*
S20	JALPLH000000000	raw poultry	Corvallis	1541	*aac(6’)-Iaa, qnrS1, floR*	-	*T57S*
S21	JALPKX000000000	raw poultry	Kentucky	198	*aac(3)-Id, aac(3)-Iid, aac(6’)-Iaa, aadA17, aadA7, aph(3’)-Ia, rmtB, blaCTX-M-55, blaTEM, qnrS1, tet(A), sul1, floR, ARR-2, dfrA14, qacE, mph(A), fosA3, lnu(F)*	*S83F, D87N*	*T57S, S80I*
S22	JALPKW000000000	raw poultry	Anatum	64	*aac(6’)-Iaa*	-	*T57S*
S23	JALPKZ000000000	raw poultry	Infantis	32	*aac(3)-IV, aac(6’)-Iaa, aadA1, aph(4)-Ia, blaCTX-M-65, tet(A), sul1, floR, dfrA14, qacE*	*D87Y*	*T57S*
S24	JALPKY000000000	raw poultry	Javiana	9074	*aac(6’)-Iaa*	-	*T57S*

* Represents consistency of all samples in the table. ST: sequence type, QRDR: Quinolone resistance determining region.

**Table 2:** Plasmid and I integron of 24 *Salmonella* isolates.

Sample	Serotype	Plasmids	Gene Found on Plasmids	Presence of Class I Integron	Gene Cassette Found on Integron
S1	London	IncFIB(K)	-	1	*aadA16, dfrA27, ARR-3* *,* *aac(6’)-Ib-cr*
S2	London	IncFIB(K)	-	1	*aadA16, dfrA27, ARR-3* *,* *aac(6’)-Ib-cr*
S3	London	IncFIB(K), IncI1-I(Alpha)	-	1	*aadA16, dfrA27, ARR-3* *,* *aac(6’)-Ib-cr*
S4	London	IncFIB(K)	-	1	*aadA16, dfrA27, ARR-3* *,* *aac(6’)-Ib-cr*
S5	Typhimurium	IncHI2A, IncHI2, IncFIB(S), IncFII(S)	-	-	-
S6	Typhimurium	IncFIB(S), IncFII(S)	-	-	-
S7	Typhimurium	IncFIB(S), IncFII(S)	-	-	-
S8	Typhimurium	IncFIB(S), IncFII(S)	-	-	-
S9	Typhimurium	IncQ1	*sul2*, *aph(6)-Id*, *aph(3’’)-Ib*	-	-
S10	Typhimurium	IncHI2A, IncHI2, IncFIB(S), IncFII(S), IncQ1	-	CALIN	*aac(6’)-Ib-cr*
S11	Typhimurium	IncFIB(S), IncFII(S)	-	-	-
S12	Goldcoast	-	-	-	-
S13	Goldcoast	-	-	-	-
S14	Liverpool	-	-	-	-
S15	Liverpool	-	-	-	-
S16	Liverpool	-	-	-	-
S17	Liverpool	-	-	-	-
S18	Liverpool	-	-	-	-
S19	Meleagridis	IncFIA(HI1), IncFIB(K)	-	-	-
S20	Corvallis	-	-	-	-
S21	Kentucky	-	-	-	-
S22	Anatum	IncFII(p96A), Col(pHAD28), Col440II	-	-	-
S23	Infantis	IncFIB(pN55391)	-	1	*aadA1*
S24	Javiana	-	-	-	-

## Data Availability

The original contributions presented in this study are included in the article. Further inquiries can be directed to the corresponding author.

## Supplementary Materials

The following supporting information can be downloaded at: https://www.mdpi.com/article/10.3390/microorganisms12112166/s1, Table S1: Quality statistical table of genomic data of *Salmonella*; Table S2: Virulence genes; Table S3: Figure 3 Supplementary data; Table S4: Figure 4 Supplementary data; Table S5: Sequencing Primer Information.
