# Supplementary material for: Whole-Genome Sequencing Analysis of Antimicrobial Resistance, Virulence Factors, and Genetic Diversity of Salmonella from Wenzhou, China
Source: Microorganisms. 2024 Oct 27;12(11):2166. doi: 10.3390/microorganisms12112166 (PMC11596050; doi:10.3390/microorganisms12112166)
Supplement: Supplementary file 1 [file microorganisms-12-02166-s001.zip › Table.S1.pdf]

Table. S1. Quality statistical table of genomic data of *Salmonella*

Sample name	Genome size(bp)	Contig number	GC content(%)	Q20(%)	Q30(%)	coverage depth (x)	N50	CDS	tRNA	rRNA
S1	4,721,537	52	52.75	97.4	92.68	228	225,411	4393	64	3
S2	4,754,089	50	52.66	97.24	92.45	241	276,266	4438	70	3
S3	4,792,994	52	52.64	97.55	93.12	309	228,760	4484	62	3
S4	4,733,533	56	52.63	97.26	92.47	230	252,253	4405	65	3
S5	5,079,122	82	52.48	97.54	92.92	193	201,941	4820	71	3
S6	4,924,688	66	52.74	97.46	92.81	234	222,696	4658	65	3
S7	4,924,460	68	52.66	97.58	93.17	235	196,368	4659	67	3
S8	4,873,985	69	52.68	97.5	92.88	221	223,194	4598	67	3
S9	5,007,606	239	52.55	97.35	92.66	201	73,840	4729	68	4
S10	5,291,259	137	52.36	97.35	92.63	273	223,581	5054	69	3
S11	4,881,193	59	52.58	97.32	92.53	205	258,689	4600	67	3
S12	4,643,475	40	52.62	97.26	92.48	243	251,504	4292	70	3
S13	4,780,053	240	52.62	97.32	92.63	233	226,572	4391	71	3
S14	4,747,530	32	52.67	97.49	92.9	224	399,791	4408	69	2
S15	4,748,110	33	52.73	97.66	93.29	220	400,394	4405	70	2
S16	4,747,791	32	52.68	97.23	92.42	233	400,394	4411	70	2
S17	4,748,071	33	52.7	97.11	92.16	250	400,394	4408	70	2
S18	4,747,754	33	52.83	97.38	92.65	243	399,791	4406	70	2
S19	4,962,640	50	52.51	97.23	92.41	227	428,274	4681	67	3
S20	4,914,792	43	52.46	97.31	92.59	225	534,429	4622	72	3
S21	4,854,425	64	52.62	97.43	92.78	247	354,097	4540	66	3
S22	4,880,030	77	52.29	97.57	93.05	214	302,101	4607	66	3
S23	4,976,146	63	52.65	97.35	92.61	209	186,897	4664	68	3
S24	4,579,495	41	52.5	97.46	92.81	300	281,846	4272	74	4

Q20 (%):the percentage of bases with the base recognition accuracy rate of more than 99%; Q30 (%): the percentage of bases with base recognition

accuracy of 99.9% or more; N50: Arrange all sequences in order of length from longest to shortest, add the lengths of the sequences in that order, and when the summed length reaches 50% of the total length of the sequence, the length of the last sequence (bp).
